# Green extraction of hemp (*Cannabis sativa* L.) using microwave method for recovery of three valuable fractions (essential oil, phenolic compounds and cannabinoids): a central composite design optimization study

**DOI:** 10.1002/jsfa.11971

**Published:** 2022-05-18

**Authors:** Eugenia Mazzara, Riccardo Carletti, Riccardo Petrelli, Ahmed M Mustafa, Giovanni Caprioli, Dennis Fiorini, Serena Scortichini, Stefano Dall'Acqua, Stefania Sut, Sonia Nuñez, Victor López, Valtcho D Zheljazkov, Giulia Bonacucina, Filippo Maggi, Marco Cespi

**Affiliations:** ^1^ School of Pharmacy University of Camerino Camerino Italy; ^2^ School of Science and Technology University of Camerino Camerino Italy; ^3^ Department of Pharmaceutical and Pharmacological Sciences, Natural Product Laboratory University of Padua Padua Italy; ^4^ Department of Pharmacy, Faculty of Health Sciences Universidad San Jorge Zaragoza Spain; ^5^ Crop and Soil Science Department Oregon State University Corvallis Oregon USA

**Keywords:** terpenes, cannabidiol, desirability, enzyme inhibition, antioxidant

## Abstract

**BACKGROUND:**

Solvent‐free microwave‐assisted extraction (MAE) is a green extraction method capable of boosting the yield and quality profile of hemp essential oil when compared with other conventional extraction techniques. During this process, two by‐products are produced, namely the aqueous residue containing bioactive phenolics and the residual deterpenated biomass, which can be used for further extraction and purification of phytocannabinoids. To date, the hemp industry has not utilized these products, although they can be valuable for the food, cosmetic, nutraceutical and pharmaceutical market.

**RESULTS:**

This study assessed and optimized the variables affecting MAE efficiency, namely microwave irradiation power, extraction time and added water, which were studied using a central composite design approach, and results were used to optimize the extraction process for recovering three valuable fractions: essential oil, polyphenols and phytocannabinoids. The products obtained using the optimized conditions were characterized in terms of yield, chemical profile and antioxidant potential. Moreover, the by‐products obtained during the optimized run were further analyzed in terms of their biological activity using both enzymatic and non‐enzymatic assays. The aqueous residue demonstrated a powerful α‐glucosidase inhibition, a good activity in terms of superoxide radical scavenging activity, a modest efficacy in terms of inhibition of advanced glycation end products formation and no activity in terms of lipase inhibition. The residual deterpenated biomass did not possess significant biological activity.

**CONCLUSION:**

This work demonstrated valorization of industrial hemp essential oil and its by‐products, obtained by a sustainable and eco‐friendly extraction method, through an almost waste‐free approach. Cannabinoids as well as other valuable bioactive compounds such as glycosidic flavones may be recovered from the residues of the essential oil extraction, representing interesting substances in the pharmaceutical, cosmetic and nutraceutical fields. © 2022 The Authors. *Journal of The Science of Food and Agriculture* published by John Wiley & Sons Ltd on behalf of Society of Chemical Industry.

## INTRODUCTION

Industrial or fiber hemp (*Cannabis sativa* L.) is an eco‐friendly crop known for its ability to sequester CO_2_ from the atmosphere, to take up and accumulate pesticides and heavy metals from contaminated soils, and to enrich the soil in organic carbon (>10 t ha^−1^), thus being an ideal crop for sustainable agriculture.^1^ Nowadays, hemp is employed on an industrial level to make textiles, paper, food, cosmetics, medicine, green building, biofuels and bioplastics. From a pharmaceutical perspective, the world hemp market has steadily increased in the last 5–10 years, boosted by the therapeutic applications of cannabidiol (CBD) and other minor cannabinoids. The world CBD market, as more generally the industrial (legal) cannabis market (industrial and medical use of inflorescences), has grown at a rate of 35–40% per year since 2016, and has doubled from 2016 to 2020. As a consequence, hemp is currently cultivated in almost 50 countries around the world. This previously unseen market expansion represents a great opportunity for the stakeholders involved in the production chain to exploit this crop's full potential in terms of production and the development of new products. In this respect, hemp essential oil (EO) may represent a niche overlooked product with potential applications in the pharmaceuticals, nutraceuticals, cosmetics and pest control industries.^2^, ^3^ Indeed, the extracted EO from industrial hemp can meet the increasing demand for oily extracts from cannabis.

Hemp EO consists of two main fractions: monoterpenes, containing mainly myrcene, α‐pinene and terpinolene; and sesquiterpenes, mainly represented by (*E*)‐caryophyllene and α‐humulene. Non‐psychotropic cannabinoids, mostly represented by CBD, may also be present in the hemp EO.^4^ The composition of the EO, however, varies depending on factors such as the crop variety, the part used (e.g., leaves vs. inflorescences), the harvest period, the state of the processed material (e.g., fresh vs. dried) and the extraction technique (e.g., conventional distillation vs. more advanced techniques), making its chemical profile highly flexible according to the final destination of this product.^4^, ^5^ Recently, more advanced techniques for the extraction of EOs have been studied to maximize the recovery and modulate the chemical profiles. Microwave‐assisted extraction (MAE) is one of these advanced methods. It relies on water heating generated by microwaves into the vacuole of plant cells, resulting in volatilization of low‐boiling‐point molecules that can then be recovered through a condenser apparatus.^6^ Notably, MAE is significantly more effective than conventional hydrodistillation (HD), allowing higher or similar yields with lower extraction times and energy and water consumption.^7^

During MAE of hemp inflorescences, an aqueous residue and the deterpenated plant biomass remain in the reactor; they could represent an important source of flavonoids^8^ and phytocannabinoids, respectively. In this respect, research on their potential for reuse on an industrial level is limited. Therefore, the goal of the present work was to optimize MAE in order to ensure high‐quality products, namely EO, an aqueous extract rich in polyphenols, and the residual biomass to be used as a source of phytocannabinoids; these products have potential application in various industries such as food, cosmetics, nutraceuticals, and pharmaceuticals. The effects of different extraction conditions applied have been evaluated through a central composite design (CCD) approach. This statistical tool is useful to rationalize the work and objectively evaluate the results by analyzing the variables incident in the process at the same time. The variables microwave irradiation power (MP), extraction time (ET) and added water into the reactor (W%) have been selected as the most influential on the process, and are the subject of this study.

The qualitative and quantitative composition of the three fractions obtained, namely EO, polyphenols and phytocannabinoids, was analyzed by gas chromatography–flame ionization detection (GC‐FID), gas chromatography–mass spectrometry (GC‐MS) and high‐performance liquid chromatography–diode array detection–mass spectrometry (HPLC‐DAD‐MS) techniques. Furthermore, the total phenolic and flavonoid content, and the 2,2‐diphenyl‐1‐picrylhydrazyl (DPPH) radical scavenger activity of the water residue extracts, were determined by spectrophotometric methods. Cannabinoids and cannabis extracts have long been studied and used for their analgesic and anti‐inflammatory properties, as well as central nervous system effects; nevertheless, little is known about its potential application in metabolic disorders such as obesity and diabetes. As *C. sativa* is a source of phenolic compounds, the ability of these extracts to interact with physiological targets involved in weight and glycemic control was explored in this study; the inhibitory enzyme properties of the extracts obtained using the MAE optimized conditions were evaluated on α‐glucosidase, lipase and xanthine oxidase, and for inhibition of advanced glycation end products (AGE) formation to support the potential of these by‐products on an industrial level.

## MATERIAL AND METHODS

### Plant material

The monoecious inflorescences of *C. sativa* cv. Futura 75 were obtained from a cultivation field sited in Fiuminata (central Italy; 43° 10′ 40′′ N, 12° 56′ 59′′ E; 451 m above sea level) and harvested at the beginning of August 2020. The codex CAME#27834 was used to archive a voucher specimen in the *Herbarium Camerinensis* of the School of Biosciences and Veterinary Medicine, University of Camerino, Italy. The samples consisted of 20–30 cm inflorescences with leaves and upper branches.

During sampling, the authors were very careful to keep samples as uniform as possible in terms of the quantity of flowers, leaves and stems. The fresh hemp samples were immediately frozen at −20 °C once received and stored until use.

### Water content determination of frozen hemp

The moisture content was determined on three samples of Futura 75 frozen inflorescences randomly collected (about 2.5 g each), after heating at 100 °C using a thermo balance (Scaltec SMO 01, Scaltec instruments GmbH, Heiligenstadt, DE). The average moisture content of the biomass was 71.3 ± 0.8%.

### Microwave‐assisted extraction

**Figure 1:**
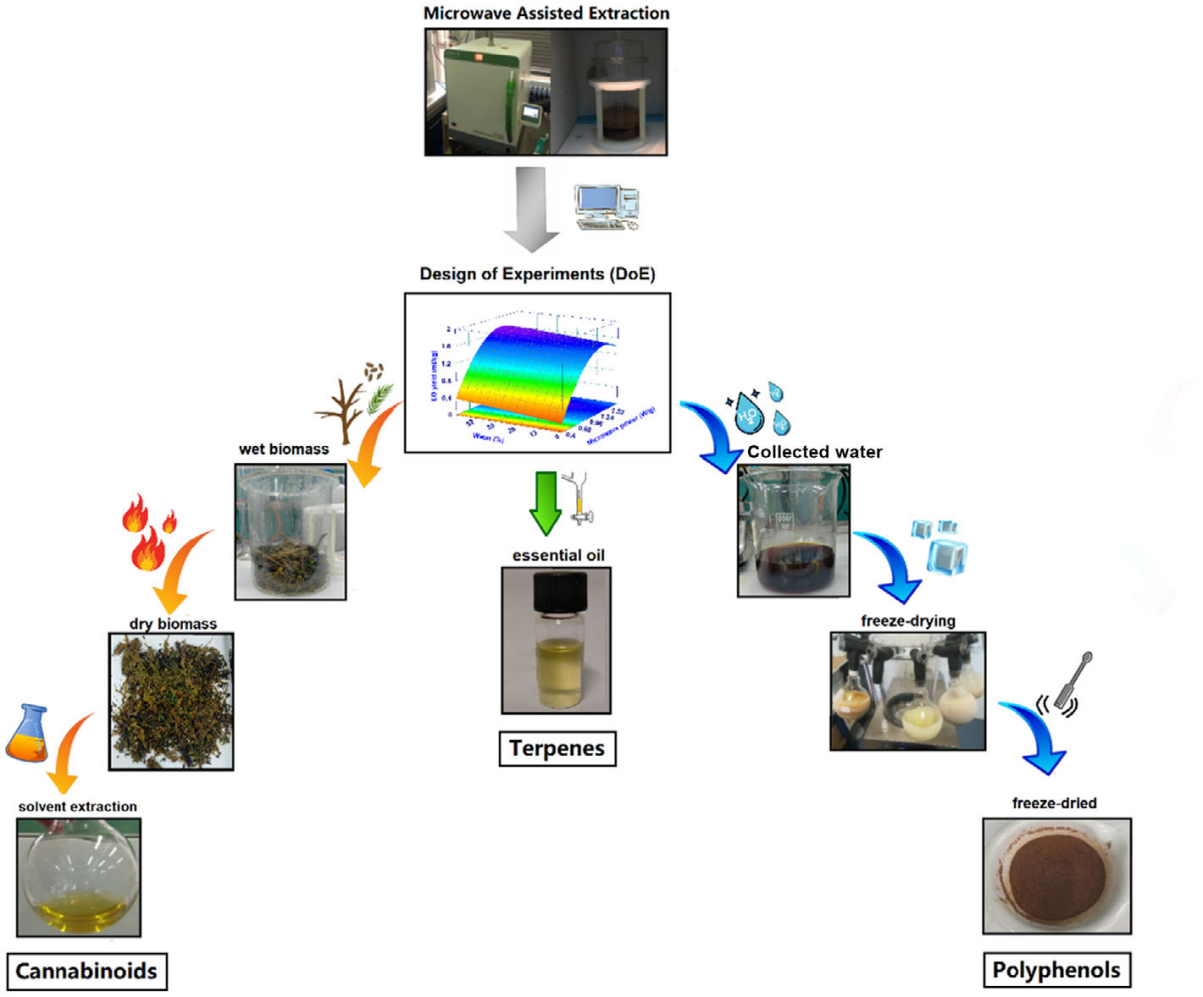
Graphical representation of the three products obtained after MAE (i.e. essential oil, aqueous residue and residual deterpenated biomass).

The MAE to obtain EO was carried out using an advanced microwave extraction system (ETHOS X, Milestone, Italy). The apparatus is composed of a microwave reactor of 2.45 GHz, equipped with an infrared sensor monitoring the temperature, and two magnetrons with a maximum delivery power of 1800 W (2 × 900 W). All the extractions were carried out using a glass reactor (Pyrex) of 5 L capacity closed with a glass cover, at atmospheric pressure. The system was equipped with a Clevenger‐type apparatus above the oven, made of stainless steel (‘Fragrances set‐up’), and, in addition, a Chiller Smart H150‐2100S, purchased from Labtech srl (Sorisole, Bergamo, Italy), was used to maintain the water temperature at 8 °C. Eighteen different experiments were carried out, varying the operative conditions, namely microwave irradiation power (MP, W g^−1^), extraction time (ET, min) and water added to the reactor along with hemp (W%), as reported in the ‘Design of the experiments (DoE)’ section. The EO, once separated from the water layer, was collected in glass vials sealed with PTFE–silicone septa and then stored at 4 °C until further analysis. At the end of MAE, two additional products were collected from the reactor: aqueous residue and deterpenated biomass. The aqueous residue was collected immediately after MAE, filtered through filter paper and stored at –20 °C until further analysis. Finally, the dual biomass (deterpenated) in the reactor was collected and dried at 60 °C using a Biosec desiccator (Tauro Essiccatori, Vanzo Nuovo, Vicenza, Italy) for around 24 h, until no weight loss was observed, and afterwards it was stored in the dark at room temperature (Fig. 1).

### Design of the experiments (DoE)

A response surface methodology, CCD, was applied to evaluate the effect of the MAE extraction conditions (MP, ET and W%) on the yield and features of all the obtained products, namely EO, aqueous residue and residual biomass. The selected design required 18 experimental runs, composed of 2^3^ factorial points (designated by the coded variables −1 or 1), 2 × 3 axial points (designated by the coded variables −1.682 or +1.682) and four replications of the central point (designated by the coded variable 0).^9^ The choice of this composite design allowed us to obtain a spherical experimental domain as well as spherical isovariance lines on the surface (rotatability), also assuring uniform precision within the experimental domain.^9^

All the experimental runs and their operative conditions are described in Table 1. All the extraction runs refer to the processing of 1 kg of the moist matrix (the relative amount of fresh plant and water varied from run to run according to the parameter W% in Table 1). The MP was set in the range of 0.7–1.5 W g^−1^ in order to obtain a maximum absolute value of 1.77 W g^−1^ (axial points at +1.682), which, for the amount of product processed (1 kg), corresponding to 1770 W, a value close to the highest limit of the instrument (1800 W). The duration of the extraction process (parameter ET) was set based on the results of a previous work on MAE.^5^

**Table 1:** Experimental conditions both in uncoded and coded variables of the 18 runs carried out according to the screening design^a^

Run	Coded variables			Uncoded variables			Absolute values		
	MP	ET	W	MP (W g^−1^)	ET (min)	W (%)	MP (W)	Water added (g)	Fresh hemp added (g)
1	−1	−1	−1	0.7	80	13	700	130	870
2	1	−1	−1	1.5	80	13	1500	130	870
3	−1	1	−1	0.7	140	13	700	130	870
4	1	1	−1	1.5	140	13	1500	130	870
5	−1	−1	1	0.7	80	50	700	500	500
6	1	−1	1	1.5	80	50	1500	500	500
7	−1	1	1	0.7	140	50	700	500	500
8	1	1	1	1.5	140	50	1500	500	500
9	−1.68	0	0	0.43	110	31.5	427	315	685
10	1.68	0	0	1.77	110	31.5	1773	315	685
11	0	−1.68	0	1.1	59.5	31.5	1100	315	685
12	0	1.68	0	1.1	160.5	31.5	1100	315	685
13	0	0	−1.68	1.1	110	0.4	1100	4	996
14	0	0	1.68	1.1	110	62.6	1100	626	374
15	0	0	0	1.1	110	31.5	1100	315	685
16	0	0	0	1.1	110	31.5	1100	315	685
17	0	0	0	1.1	110	31.5	1100	315	685
18	0	0	0	1.1	110	31.5	1100	315	685

^a^ In the final columns are also reported the absolute values of total microwave power applied and the amount of water and plant added in the microwave reactor.

From each extraction run three different products were obtained (Fig. 1):

- EO, which was evaluated in terms of:

- Yield
(1) $$\mathrm{EO}\;\text{yield}\;\left(\%\right)=\frac{\text{Weight of}\;\mathrm{EO}\;\left(g\right)}{\text{Weight of}\;\mathrm{dry}\;\text{biomass}\;\left(g\right)}\times 100$$
- Content of volatile main constituents (g 100 g^−1^ EO), acquired by GC‐FID as described in the ‘GC‐FID analysis’ section below.

II Lyophilized aqueous extract (AE) (‘Sample treatment’ section, below), characterized in terms of:

- Yield
(2) $$\mathrm{AE}\;\text{yield}\;\left(\mathrm{mg}\;100\;g^{-1}\right)=\frac{\text{weight of liophilized water residue}\;\left(\mathrm{mg}\right)}{\text{weight of fresh biomass}\;\left(g\right)}\times 100$$
- Total polyphenol content (determined as reported in the ‘Total polyphenol content’ section, below)
- Total flavonoid content (determined as reported in the ‘Total flavonoid content (TFC)’ section, below)
- Antioxidant activity (determined as reported in the ‘Antioxidant activity’ section, below).

III Residual biomass hexane extract (HE), characterized in terms of:

- Concentration of the main phytocannabinoid, cannabidiol (g 100 g^−1^ dry biomass), determined as reported in the ‘GC‐FID analysis of the hexane extracts (HE)’ section, below.

### Analysis of EOs

#### Density determination

The density of the 18 EOs obtained by MAE was determined using a digital density meter with an oscillating U‐tube (DA‐100M, Mettler Toledo) at 20 °C. The obtained mean density value was 0.883 g mL^−1^ ± 0.002.

#### GC‐FID analysis

Eleven EO components, namely α‐pinene, β‐pinene, myrcene, limonene, 1,8‐cineole, (*E*)‐β‐ocimene, terpinolene, (*E*)‐caryophyllene, α‐humulene, caryophyllene oxide and CBD were analyzed in the EOs by GC coupled with FID. The analytical standards of these compounds were purchased from Sigma‐Aldrich (Milan, Italy) and were used to prepare the calibration curves in the range 0.005–10 mg mL^−1^. Before injection, 6 μL hemp EOs was diluted in 594 μL *n*‐hexane (LC‐MS) and 0.5 μL injected in split mode (1:30) for the analysis. An Agilent 6850 gas chromatograph equipped with an HP‐5 coated capillary column (HP‐5, 30 m length, 0.32 internal diameter, 0.25 film thickness; Agilent Technologies) was used. The injector temperature was 300 °C and hydrogen was the carrier gas, produced with a generator (PGH2‐250, DBS Analytical Instruments, Vigonza, Italy). The gas flow was set at 3.7 mL min^−1^. The total run time was 15.60 min. Specifically, the gas chromatograph oven temperature was held at 60 °C for 3 min, then the temperature was raised to 350 °C at 25 °C min^−1^ and held for 1 min. The temperature of the flame ionization detector was 360 °C, with a hydrogen and air flow of 40 and 400 mL min^−1^, respectively.

#### GC‐MS analysis

For qualitative analysis of the EO chemical composition, an Agilent 6890N GC‐MS system coupled with a 5973N single‐quadrupole detector and a 7863 autosampler (Agilent, Wilmington, DE, USA) was employed. A capillary HP‐5MS column was used (5% phenylmethylpolysiloxane, 30 m length, 0.25 mm internal diameter, 0.1 μm film thickness; Agilent). The temperature of the oven was held at 60 °C for 5 min, then ramped up at 4 °C min^−1^ until 220 °C and finally ramped at 11 °C min^−1^ to 280 °C. The flow rate of the carrier gas He (99.5%) was set at 1 mL min^−1^. Once diluted in *n*‐hexane (LC‐MS) 1:100, the EOs were injected in split mode with an electron energy of 70 eV. The major EO compounds were identified by co‐injection of analytical standards, whereas the other constituents were found by comparing retention indices (RIs) and mass spectra to those reported in the literature.^10^, ^11^, ^12^

### Analysis of lyophilized aqueous extract (AE)

#### Sample treatment

The frozen samples were dried to constant weight using a BUCHI Lyovapor L‐200 freeze‐dryer (Büchi Labortechnik AG, Flawil, Switzerland) at −54 °C, with a pressure of 0.05 mbar and a shelf temperature of 10 °C. The dried samples were then ground using a mortar and pestle to give a fine brownish powder. Dried powders were sealed and stored at 4 °C until analysis.

#### Total polyphenol content

The Folin–Ciocalteu method described previously^13^ was used for evaluation of total polyphenol content (TPC), with some modifications as follows: 0.5 mL aqueous extract solutions at a concentration of 1 mg mL^−1^ were placed in test tubes, and 2.5 mL Folin–Ciocalteu reagent solution (diluted ten times in water) and 7 mL of 7.5% Na_2_CO_3_ solution were mixed. Then, the test tube containing the reaction mixture was kept in the dark at room temperature for 2 h and absorption of the sample was measured spectrophotometrically at 735 nm using a Cary 8454 UV–visible spectrophotometer (Agilent Technologies, Woburn, MA, USA). A gallic acid calibration curve was constructed and used for quantification of TPC in the AE. The obtained results were calculated as the average of two experiments and the TPC was expressed as milligrams of gallic acid equivalents (GAE) 100 g^−1^ dry extract (DE).

#### Total flavonoid content (TFC)

TFC was determined according to Chen *et al*.,^14^ with slight modifications as follows: 0.5 mL aqueous extract solutions at a concentration of 1 mg mL^−1^ were mixed with 0.15 mL NaNO_2_ (0.5 mol L^−1^), then 3.2 mL of 30% methanol and 0.15 mL of 0.3 mol L^−1^ AlCl_3_.6H_2_O were added with shaking. After 5 min, 1 mL of 1 mol L^−1^ NaOH was added. Then, after mixing the solution, the absorbance was recorded at 506 nm against the blank reagent using a UV–visible spectrophotometer (Cary 8454, Agilent). A rutin calibration curve was made using different concentrations (100–1000 ppm) under the same conditions. TFC was expressed as milligrams of rutin equivalents (RE) 100 g^−1^ dried extract (DE). Analyses were performed in duplicate.

#### Antioxidant activity

The antioxidant activity of AE was estimated spectrophotometrically against DPPH free radical according to the method described by Mustafa *et al*.^13^ The procedures were as follows: 0.5 mL of AE aqueous solution was mixed with 4.5 mL of 0.1 mmol L^−1^ DPPH (dissolved in ethanol). The mixture was incubated for 30 min at room temperature in the dark and, finally, DPPH color disappearance was recorded spectrophotometrically (Cary 8454, Agilent) at 517 nm. The calibration curve of Trolox as the reference antioxidant was constructed, and the results were calculated as milligrams of Trolox equivalents (TE) per kilogram of DE. Experiments were performed in duplicate.

### Residual biomass HE analysis

#### Sample pretreatment and extraction

The residual biomass samples from the 18 extraction runs were preliminarily treated and extracted as reported in the THC determination procedure published by the European Commission.^15^ Briefly, after drying, the stems and seeds larger than 2 mm were removed, while the remaining material was reduced to particles with sizes less than 1.0 mm using an electric mill (MFC, IKA‐Werk, Staufen, Germany). Then, 100 mg dry hemp powder was mixed with 5 mL analytical‐grade *n*‐hexane (Sigma‐Aldrich) and extracted at room temperature for 20 min in an ultrasound bath (AU‐220, Argo Lab, Carpi, Italy). After 10 min centrifugation at 5000 rpm, the supernatant (HE) was separated and dried with MgSO_4_.

#### GC‐FID analysis of HE

CBD quantification in HE from residual biomass was performed according to the European Commission.^15^ Briefly, 0.5 μL samples were injected into the GC‐FID equipment and analyzed as previously reported for the EOs (‘GC‐FID analysis’ section, above). For comparative purposes, the same analysis was performed on the fresh biomass not previously subjected to MAE.

### DoE analysis

The results of each single response for all 18 runs of the CCD were analyzed by multilinear regression using a full quadratic model (Eqn (3)):

(3) $$y=\beta_{0}+\sum_{i=1}^{n}\beta_{i}x_{i}+\sum_{i=1}^{n}\beta_{\mathrm{ii}}x_{i}^{2}+\sum_{i<j}\beta_{\mathrm{ij}}x_{i}x_{j}$$

where *y* is the response, *β*_0_ is the model constant, *β*_*i*_ is the coefficient corresponding to the variables *x*_*i*_ (linear terms), *β*_*ii*_ are the coefficients associated with the variables *x*_*ii*_ (quadratic term) and *β*_*ij*_ are the coefficients associated with the variables *x*_*ij*_ (first‐order interaction terms). All the full quadratic models were then subjected to a variable selection procedure (model reduction) to improve the precision of the estimated coefficients of the retained variables, minimize the mean square error and, more generally, satisfy the principle of parsimony.^16^, ^17^ Stepwise regression operating in backward elimination mode was used to perform the model reduction process. The adjusted coefficient of multiple determination (*R* ^2^_adj_), the predicted coefficient of multiple determination (*R* ^2^_pred_) and the Mallows’ Cp statistic were the parameters used to choose the best model among all those obtained from the stepwise regression.^17^ Analysis of variance (ANOVA), coefficient and residual analysis were used to evaluate all the final models. The model fitting, reduction, selection and analysis were performed with Minitab 18 evaluation statistical software.

### Optimization and validation of MAE process

All the models defined from the DoE analysis were used to optimize the MAE process, in order to identify the best experimental conditions to provide satisfactory results for all the selected responses for the three obtained products (EO, AE and HE) at the same time. Multiple responses optimization was carried out by means of the desirability technique.^18^, ^19^ A partial desirability function (Dp) aimed to maximize the responses was chosen for all the responses. The composite desirability function D was calculated as the geometric mean of all the Dp for all the combinations of the three investigated parameters, i.e. MP, ET and W%. D ranges between 0 (at least one response is completely unsatisfactory) and 1 (all the responses are completely satisfactory). A surface map of D was built and used to identify the regions where D was the highest (closest to 1). Two different sets of experimental conditions (V1 and V2) were identified, and the predicted responses, as well as their 95% prediction intervals, calculated. The runs V1 and V2 were performed, and the obtained results compared with the desirability predictions.

### Characterization of products from the MAE optimized runs

Products (i.e. EO, AE and HE) from V1 and V2 MAE runs (optimized runs) were characterized for all the parameters used for DoE (‘Design of the Experiments (DoE)’ section to ‘Residual biomass HE analysis’ section, above).

#### LC‐DAD‐MS^*n*^ analysis of AE and HE

The AE and HE from the best extraction (V1) were further analyzed using an HPLC‐DAD‐MS^*n*^ system. Briefly, samples were weighed and extracted (20 mg mL^−1^ methanol) using an ultrasound bath for 10 min. Samples were centrifuged at 13 000 rpm for 15 min, and the supernatants were used for LC analysis. The LC‐MS^*n*^ system consisted of an Agilent 1260 quaternary pump coupled to both a 1260 Agilent diode array detector (DAD) and a Varian MS 500 mass spectrometer equipped with electrospray (ESI) ion source. A Synergi Polar‐RP 80A column (100 × 4.6 mm, 4 μm) was employed as a stationary phase. The mobile phase was a mixture of 1% formic acid in water (A) and acetonitrile (B); the gradient was set as follows: 0 min, 5% B; 30 min, 100% B; 32 min, 100% B; 32.5 min, 5% B; 34 min, 5% B. The flow rate was 0.4 μL min^−1^. Injection volume was 10 μL and the temperature was set at 30 °C. DAD allowed the collection of data in the *λ* range of 200–640 nm. Mass spectral data were acquired both in positive and negative ion mode, in the *m*/*z* range of 100–2000. The fragmentation pattern of the most intense ion species was obtained using the turbo data depending on the scanning (TDDS) function of the instrument. The MS parameters were set as follows: needle voltage 4.9 kV; shield voltage 600 V; capillary voltage 80 V; RF loading 80%; nebulizing gas pressure 25 psi (nitrogen); drying gas pressure 15 psi; drying gas temperature 300 °C. Identification of the compounds was obtained based on comparison with the literature and reference compounds when available. For compound quantification, rutin, quercetin, chlorogenic acid, CBD and cannabidiolic acid were used. Standard solutions were prepared in the concentration ranges 1–100 μg mL^−1^ and calibration curves were built. Limit of detection (LOD) and limit of quantification (LOQ) were 0.03 and 0.09 μg mL^−1^, respectively, for CBD and cannabidiolic acid.

#### Biological assays (α‐glucosidase, AGEs, lipase and superoxide radicals inhibition) of AE and HE

AE and HE from the best extraction (V1) were evaluated for biological activities through enzymatic and non‐enzymatic assays.

The capacity of hemp extracts to inhibit α‐glucosidase was measured in a 96‐well microplate reader at 405 nm.^20^ Each well contained 50 μL sample and 100 μL enzyme (1 U mL^−1^) solved in the buffer (12.5 mmol L^−1^ Na_2_HPO_4_, 3.3 mmol L^−1^ NaH_2_PO_4_; pH 6.9). After 10 min of incubation at room temperature, 50 μL pNPG (3 mmol L^−1^) was added and incubated at 37 °C for 15 min, then absorbance reading took place. Acarbose was used as the positive control.

The non‐enzymatic inhibition of AGE formation by the hemp extracts AE and HE was measured by fluorescence in 96 black well‐plates.^21^, ^22^ 50 μL BSA solution (10 mg mL^−1^), 80 μL of 0.1 mol L^−1^ phosphate buffer (containing sodium azide 3 mmol L^−1^, pH 7.4), 50 μL fructose solution (0.5 mol L^−1^) and 20 μL sample extracts (serial dilutions) were mixed. After incubating for 24 h at 37 °C, plates were analyzed at an excitation wavelength of 355 nm and emission wavelength of 460 nm. Aminoguanidine (AMG), an experimental drug used in the treatment of diabetes, was used as the positive control.

The capacity of extracts to inhibit lipase was measured in 96 well plates; 40 μL extract solution (serial dilutions) was mixed with 40 μL enzyme (2.5 mg mL^−1^ in 0.1 mol L^−1^ phosphate buffer, pH 7.0) previously centrifuged at 2000 rpm for 7 min, and 20 μL substrate solution (10 mmol L^−1^ *para‐nitrophenyl butirrate*). After incubation for 10 min at 37 °C, absorbance was read at 405 nm. Orlistat was used as the positive control.

The xanthine/xanthine oxidase assay was performed to measure the capacity of AE and HE to scavenge superoxide radicals.^23^ 90 μmol L^−1^ xanthine, 16 mmol L^−1^ Na_2_CO_3_ and 22.8 μmol L^−1^ nitro blue tetrazonium were dissolved in a phosphate buffer 18 mmol L^−1^ (pH 7) to reach a volume of 240 μL. Then, 30 μL sample and 30 μL xanthine oxidase (168 U L^−1^) were added to start the reaction. The mixture was incubated for 2 min at 37 °C. Absorbance was measured at 560 nm, and the activity of hemp extracts was determined by the transformation of NBT to the blue chromogen dye by the superoxide radical (O_2_^−^).

The inhibition for each assay was calculated using the following formula, introducing either absorbance or fluorescence as value depending on the procedure:

$$\text{Inhibition}\;\left(\%\right)=\left[\left({\text{Value}}_{\text{control}}-{\text{Value}}_{\text{sample}}\right)/{\text{Value}}_{\text{control}}\right]\times 100$$

For each assay, the IC_50_ value was calculated using nonlinear regression^24^, ^25^ (Prism version 5.0, GraphPad Inc., San Diego, CA, USA).

## RESULTS AND DISCUSSION

The present study has shown for the first time the application of statistical tools for the study and optimization of the variables involved in the extraction of fresh hemp inflorescences using MAE. Thus, all the products obtainable, namely the EO, AE and HE from residual biomass, could be industrially exploited as a valuable source of terpenes, polyphenols and phytocannabinoids, respectively. Therefore, this study paves the way for the set‐up of an extraction process with almost zero waste, where each of the obtained products represents a valuable resource.

### Analysis of EOs

**Figure 2:**
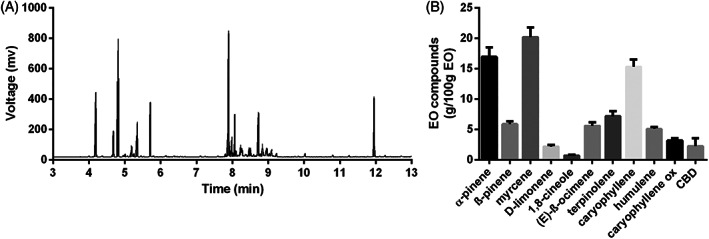
(A) Qualitative chemical profile of EO (run No. 8) obtained by FID analysis and (B) average quantitative composition of the EO extracted during the 18 MAE runs carried out according to CCD.

The EO yield (concentration) obtained using MAE was 0.412% w/w ± 0.124. The average quantitative composition of EO by the 18 runs was acquired by GC‐FID analysis and is reported in Fig. 2, along with a chromatogram showing an example of the chemical profile of the EOs (the example refers to the run No. 8). Examples of the detailed qualitative composition of EOs (from run Nos. 1, 8 and 10) and related chromatogram (run No. 8), characterized by GC‐MS analysis, are reported in Supporting Information Table S1 and Fig S1, respectively. The multiple regression (DoE analysis) of yields and concentration of EO components generated reliable mathematical models (which were able to describe the responses correctly) only for a limited number of responses, specifically the yield and the content of CBD, terpinolene, myrcene, limonene and 1,8‐cineole. All these responses were characterized by a significant regression and an *R* ^2^_adj_ > 0.58. For all the other responses, the models did not satisfactorily fit the experimental data, as demonstrated by values of *R* ^2^_adj_ < 0.5 and, in some cases, by a statistically non‐significant regression (as for (*E*)‐β‐ocimene, α‐humulene, (*E*)‐caryophyllene and caryophyllene oxide).

**Figure 3:**
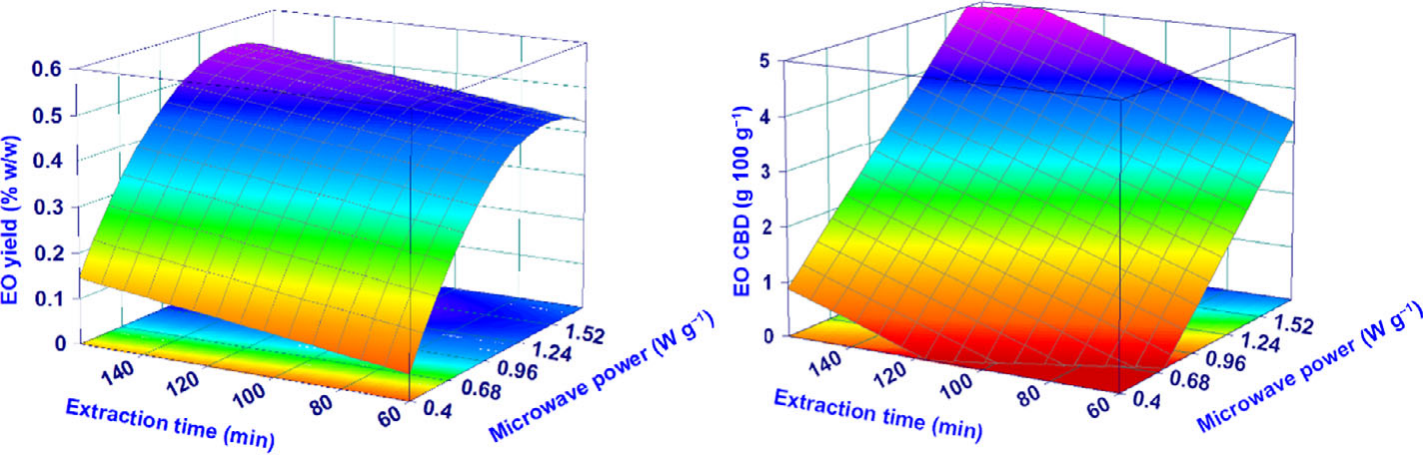
Surface plots for yield (%) and CBD concentration. The plots show the effect of microwave irradiation power and extraction time, keeping constant the added water at the coded level of 0 (the added water was never a relevant parameter for yield (%) and the CBD amount).

The models obtained by multiple regression and their evaluation parameters are reported in Table 2 and Supporting Information Table ST2. Only the responses reasonably well fitted were described. Interestingly, the goodness of how the responses are modeled is consistent with data from a previous report.^5^ In the latter, the CCD was applied to study the relevance of MAE experimental parameters on EO obtained from dry hemp biomass, confirming that only the yield and the concentration of a limited number of volatile components are sensitive to the extraction parameters. The yield and CBD content, the two responses better described by the models (*R* ^2^_adj_ > 0.72 and *P*‐value of regression < 0.001), are reported in Fig. 3 using the response surface plots. In both cases, the two responses were maximized by high values of MP, while the ET was a statistically significant parameter only for the CBD amount. These results are qualitatively comparable to those previously obtained during the MAE extraction of EO from dry hemp biomass,^5^ indicating that the experimental parameters MP and ET act in the same way independently by the hemp storage treatment (drying or freezing). However, remarkable differences can be observed from a quantitative point of view. In fact, the mean yield of EO from fresh hemp was around four times higher than those previously obtained from dry samples using the same experimental approach. This result is not surprising since an increase of EO yield of around three to eight times has been previously reported comparing fresh and dry hemp during hydro‐distillation^4^ and MAE.^26^ Another relevant point to be highlighted is the absolute value of the yield in comparison with those obtained in other studies carried out with MAE on fresh samples. In the present study, the average yield of all the runs was 0.41% ± 0.12, remarkably higher than those reported by Gunjević *et al*.^8^ (0.16–0.24%) and Micalizzi *et al*.^26^ (0.11–0.27%). Taking into account that part of such differences may derive from the different varieties of hemp compared, the reasons for such relevant differences can be explained by the experimental conditions applied. In fact, if the MP used in these works is calculated as in this study, specifically the power applied for grams of processed product (hemp plus water), the applied maximum power would result in 0.21–0.29 and 0.75 W g^−1^ for the two aforementioned studies, respectively. Here, all the 18 runs were carried out using an MP ranging from 0.43 to 1.77 W g^−1^, and the multiple regression model (Table 2) clearly indicated that the EO yield was strongly dependent on the applied power. According to the model developed in this study, a yield between 0.1% and 0.3% (the range reported in the literature) can be obtained at MP levels lower than 0.6–0.8 W g^−1^ (as a function of the extraction time), which are those effectively applied by Gunjević and Micalizzi.^8^, ^26^ To better visualize this finding, a contour plot for EO yield predicted by the model is given in Supporting Information Fig S2.

**Table 2:** Best mathematical models for some of the responses and their evaluation parameters: coefficients of determinations (*R* ^2^_adj_ and *R* ^2^_pred_), Mallows’ Cp statistic and ANOVA results (*P*‐values of regression and lack of fit)^a^

Product^b^	Response	Best model^c^	*R* ^2^	*R* ^2^_adj_	*R* ^2^_pred_	Mallows’ Cp	*P*‐value regr^d^	*P*‐value LOF^d^
EO	Yield (mL kg^−1^)	*y* = −0.347 + 1.008 MP + 0.001 ET − 0.339 MP^2^	0.776	0.728	0.253	0.82	***	ns
	Myrcene (g 100 g^−1^)	*y* = 24.46 − 10.85MP − 0.146ET − 0.070W + 0.001ET^2^ − 0.084MP*ET − 0.087MP*W + 0.002 ET*W	0.765	0.583	0.004	7.13	*	ns
	Limonene (g 100 g^−1^)	*y* = 5.196 − 2.304MP − 0.036ET + 0.020W + 1.0800MP^2^ − 0.015MP*W	0.823	0.658	0.214	8.53	*	ns
	1,8‐Cineole (g 100 g^−1^)	*y* = 1.838 + 0.071MP − 0.017ET − 0.009W − 0.004 MP*W	0.805	0.689	0.169	5.81	**	ns
	Terpinolene (g 100 g^−1^)	*y* = 3.83 − 7.96MP + 0.139ET + 5.05 MP^2^ − 0.036 MP*ET	0.804	0.716	0.652	5.65	**	ns
	CBD (g 100 g^−1^)	*y* = −4.016 + 3.218MP + 0.020ET + 0.013W	0.828	0.788	0.692	0.43	***	ns
AE	Yield (mg 100 g^−1^)	*y* = −534 + 904MP + 20.58W − 485MP2	0.892	0.870	0.779	0.29	***	ns
	TPC (mg GAE g^−1^_AE_)	*y* = 82.9 + 11.35MP − 0.141ET + 1.357W − 0.016W^2^	0.667	0.546	0.359	1.34	*	ns
	TFC (mg rutin equiv. g−1 _AE_)	*y* = 123.6 + 6.42MP − 1.525ET + 0.567W + 0.005ET^2^ − 0.010W^2^ + 0.005ET*W	0.854	0.757	0.451	4.56	**	ns
	DPPH (mg Trolox equiv. g^−1^_AE_)	*y* = −29.5 + 140MP + 0.099ET + 0.217W − 55.9MP^2^	0.748	0.657	0.389	1.61	**	ns
HE	CBD (g 100 g^−1^)	None of the tested models is able to describe the CBD amount in the biomass residue as a function of the experimental conditions applied during MAE (for all the tested models the regression was not statistically significant)						

^a^ The responses reported in this table are exclusively those having a statistically significant regression (*P*‐value regr < 0.05) and an adjusted multiple regression coefficient higher than 0.5.

^b^ EO, essential oil; AE, lyophilized aqueous extract; HE, residual biomass hexane extract.

^c^ The models are reported using the coefficients calculated from the uncoded variables.

^d^ The results of *P*‐value columns are reported as follows: ns = *P* > 0.05; *0.05 < *P* < 0.01; **0.01 < *P* < 0.001; ***P < 0.001.

The average amount of CBD in the EOs, as determined by GC‐FID analysis, was 2.24 ± 1.32 g 100 g^−1^, with a maximum value of 4.76 g 100 g^−1^ at high MP and long ET (run No. 8). In this case a comparison with literature data for EOs from dry hemp^5^ gives opposite results with respect to the yield, demonstrating that operating with fresh samples would provide a higher amount of EO but lower CBD content. Again, this result was in agreement with previous reports both for HD^4^ and MAE,^26^ and could be explained by partial evaporation of the more volatile terpene fractions during sample pretreatment (e.g., drying, grinding and storage).

Concerning the absolute values of CBD, the literature comparison is not easy due to significant differences in the utilized analytical procedures. However, when data were acquired similarly and reported as the concentration in the EO, the general results appeared similar,^26^ although the results from the latter authors were obtained using milder conditions (lower MP and shorter ET) with respect to the present study.

### AE analysis

#### Yield, TPC, TFC and antioxidant activity

**Figure 4:**
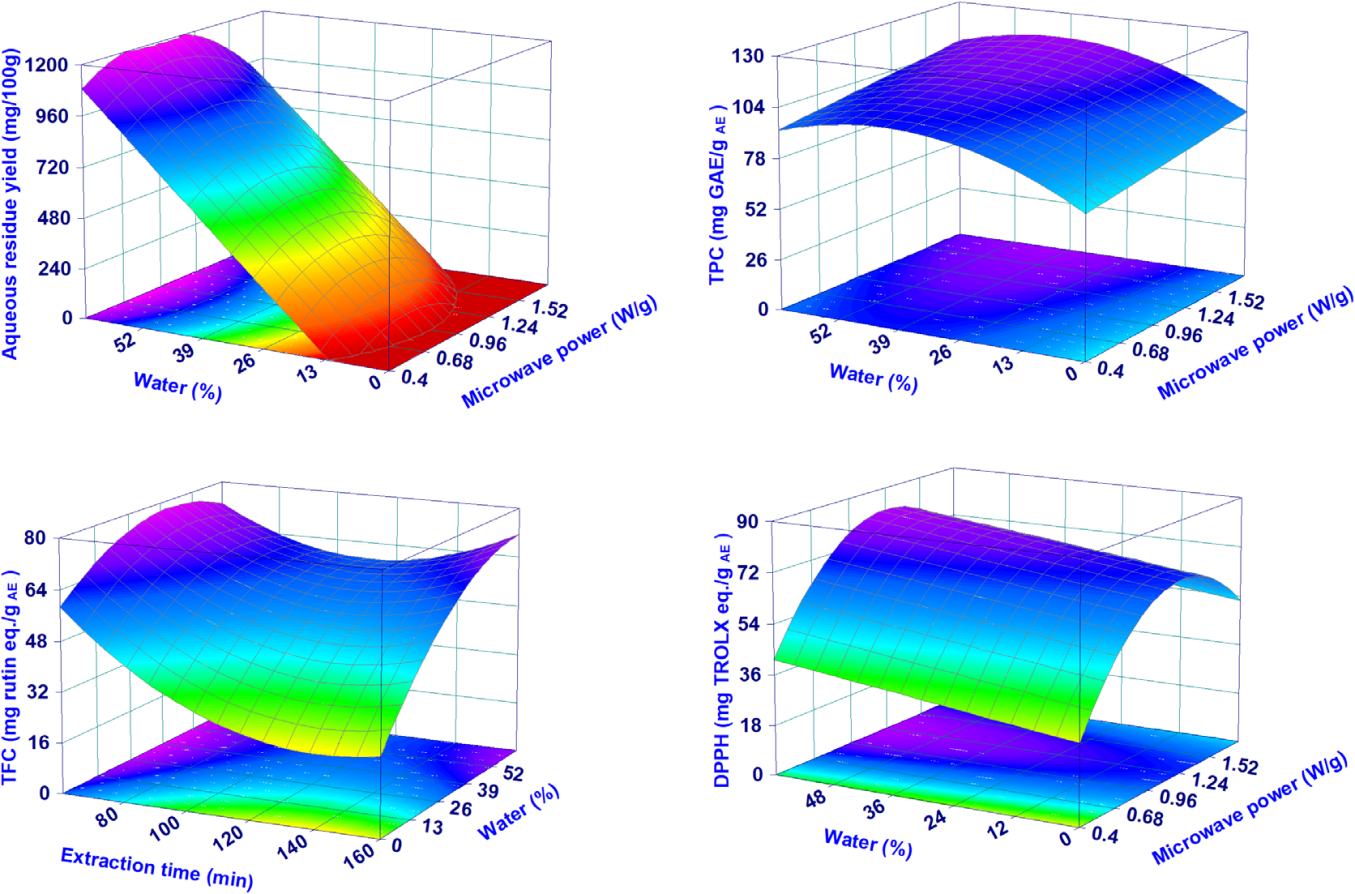
Surface plots showing the effect of the two most relevant parameters on AE yield, TPC, TFC and antioxidant capacity. In each graph, the not reported experimental parameter was always not statistically significant and, when present in the models, it was kept constant at the coded level of 0.

One of the waste products of MAE is represented by the aqueous residue, resulting mainly from the added water for the MAE process and the moisture of the fresh biomass (the water content of the fresh biomass was 71.3 ± 0.8%). As reported by previous studies, this aqueous residue should be rich in valuable water‐soluble components such as polyphenols.^27^, ^28^ The DoE analysis was performed on the process yield, the total content of phenols (TPC) and flavonoids (TFC), and DPPH radical scavenging activity. All the four responses were adequately described by the models (*R* ^2^_adj_ > 0.54 and regression was always statistically significant). The models and their evaluation parameters are reported in Table 2, while the graphical representation of the models using the surface plots is in Fig. 4.

The yield of the AE was very well described by the model, being almost completely dependent on the W%. When this was lower than 10%, the model predicted a yield equal to 0, which is consistent with the results of run Nos. 10 and 13, where no aqueous residue (and consequently no AE) was obtained. Notably, both runs were characterized by low and very low amounts of W%, respectively. Similar results were found for the TFC (W% is the main parameter), even if, in this case, the ET is a significant factor as well. On the other hand, the DPPH results depended mainly on the MP parameter, while the TPC, despite being significantly affected in a statistically significant manner by all the investigated factors, was characterized by a low variability, which likely explains the poorer regression performance compared with the other AE responses (Table 2). A comparison of these results with the literature appears difficult. In fact, despite there being two other studies devoted to the DoE analysis of microwave‐assisted liquid extraction of antioxidant compounds from cannabis,^27^, ^28^ these were focused on the extraction of antioxidants by means of organic solvents (without recovering the EO) and consequently are not relevant to the results from this study. As an example, the results of TPC reported by Drinić *et al*.^27^ are in the range of 0.8–2.7 mg GAE mL^−1^, those of Matešić at al.^28^ in the range of 5–35 mg GAE g^−1^ dry biomass, while those in the present study are in the range of 94–125 mg GAE g^−1^_AE_. The TPC results of the present study can only be compared with that of Gunjević *et al*.,^8^ who used a similar procedure although for a single sample, which is a major limitation. They obtained a value of 55 mg GAE g^−1^ lyophilized extract, which is about half of the average value (109.5 ± 9.3 mg GAE g^−1^_AE_) found in our study. According to the regression model obtained for TPC, a result of around 50 mg GAE g^−1^ lyophilized extract is obtainable operating at very low MP, which is compatible with the power value applied by Gunjević *et al*.^8^ (0.21 W g^−1^).

### Residual biomass analysis

#### The CBD content of the residual biomass

The residual biomass obtained was the remaining material after MAE extraction of the EO and removal of the aqueous residue. This residual biomass could represent a valuable source of phytocannabinoids, since MAE of EO decreases the content of this fraction only in a marginal way,^8^ whereas the aqueous residue is mainly composed of hydrophilic compounds.^27^, ^28^ No burning of the residual biomass samples was observed during the 18 MAE runs, suggesting that all the different experimental conditions applied during the CCD do not cause complete water evaporation with consequently fast temperature rise (temperature above 100 °C), sample burning and organic compound degradation.

The GC‐FID chromatogram of HE from residual biomass (run No. 8) is shown in Supporting Information Fig S3. After the 18 extraction runs the average amount of CBD still present in the residual biomass was equal to 2.35 ± 0.47 g 100 g^−1^ dry biomass. The initial CBD amount (in no MAE‐treated samples) was of 3.10 ± 0.25 g 100 g^−1^ dry biomass. Therefore, the amount of CBD still available after MAE was 75.84% ± 15.01 relative to its amount prior to MAE.

**Figure 5:**
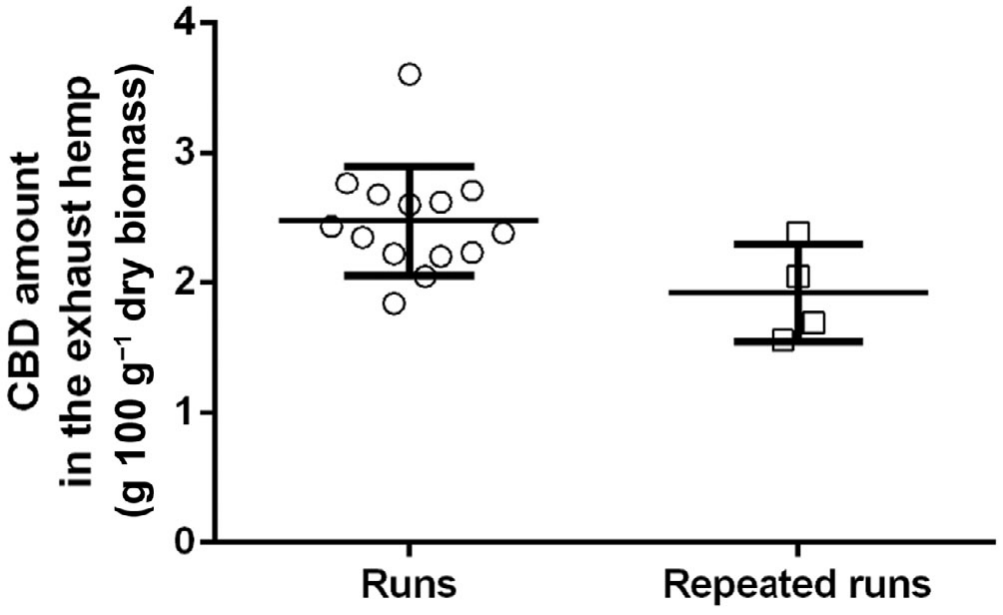
Comparison between the CBD concentration in the residual biomass of the unreplicated runs (from Nos. 1 to 13) and the replicated ones (from Nos. 15 to 18).

The multiple regression of CBD amount in the residual biomass was not statistically significant, meaning that no relationships can be found between the CBD in the residual biomass and the MAE experimental parameters. This result could be due to several factors, such as an effective absence of any relationships between response and parameters, the use of inadequate mathematical models, or an excessive variability of the results. The variability between the repeated runs (from Nos. 15 to 18) and the others was comparable (values of coefficient of variation of 19.28% vs. 17.03%) (Fig. 5). Thus the issues in multiple regression of CBD seem to be related to the intrinsic variability of CBD in the samples.

### Optimization and validation of the MAE process

The optimization process was carried out exclusively on the responses that can be efficiently modeled, and which are considered relevant for the quality of the whole MAE process, i.e. the yield of EO and its CBD content, and the yield, TPC, TFC and antioxidant efficacy of AE. All these responses varied in a different manner as a function of the experimental parameters. For example, the yield and CBD amount in the EO were not affected by the amount of the W%, while they could be maximized operating at high MP. On the other hand, the yield of the AE required high W%, while the MP had no effect at all.

**Figure 6:**
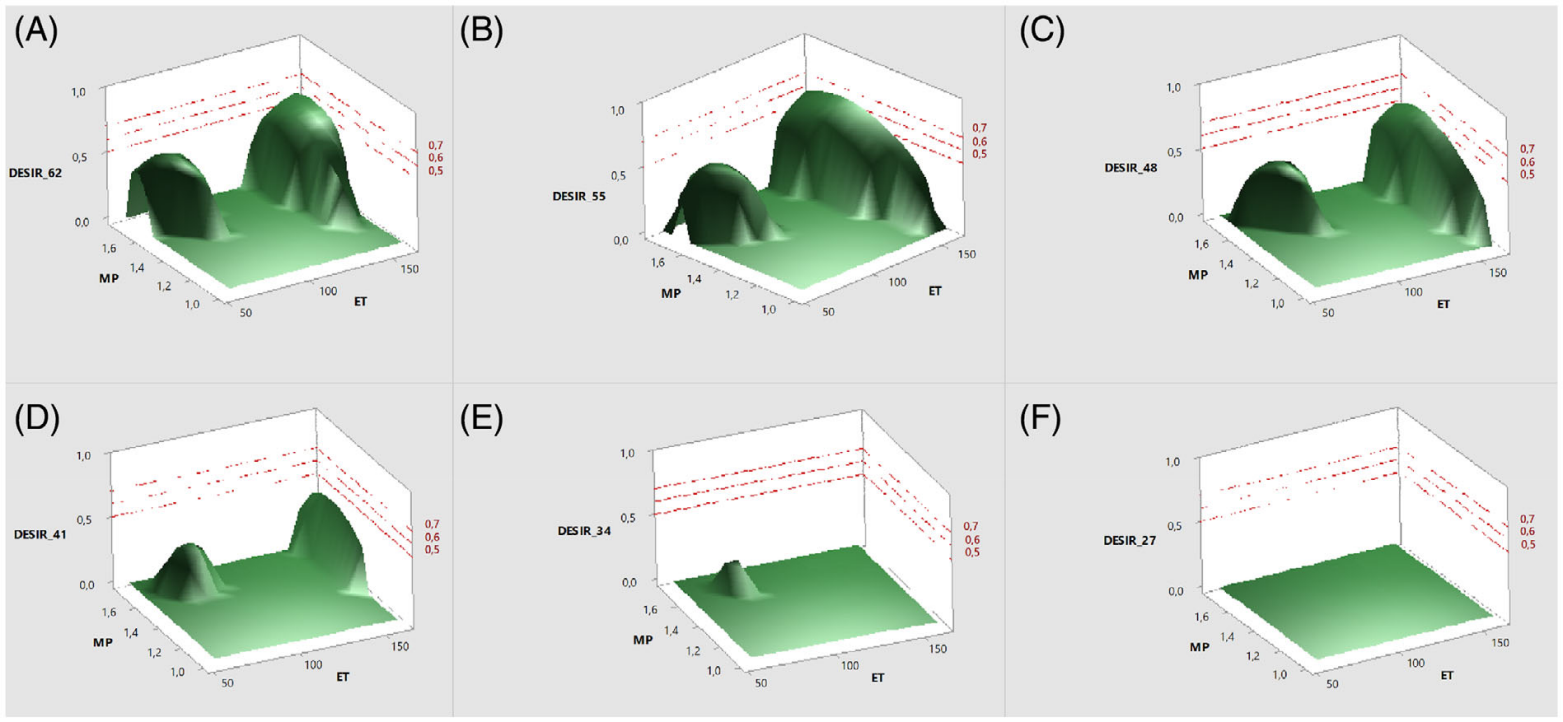
3D surface map regarding the variation of D (composite desirability) as a function of the MP, ET and W%. The W% was kept constant at a fixed level of 62%, 55%, 48%, 41%, 34% and 27% (panels A–F, respectively).

A general overview of how the experimental parameters maximize all six responses at the same time can be obtained, mapping the variation of D (composite desirability) as a function of the MP, ET and W%. Since we have three independent and one dependent variables, the W% was kept constant at a fixed level of 62%, 55%, 48%, 41%, 34% and 27% (panels A–F in Fig. 6) to obtain a 3D surface map. The surface map showed the presence of two distinct areas having a desirability higher than 0, meaning that all the considered responses were all together over the minimum level of acceptability. Interestingly, the two areas had their maximum at a high level of W% (>48%) and tended to lower and to disappear as the W% decreased. Such a behavior as a function of the water was mainly related to the yield of AE, which was strongly affected by the amount of W%. The two areas were both located at medium/high values of MP, but they differed from the length of the extraction process. From the analysis of the partial desirability function (Dp), the biggest area (medium/high MP and long ET) allowed us to satisfactorily maximize all the responses with the exception of TPC (Dp around 0.3), while the smaller zone (medium/high MP and short ET) assured poor performance in terms of TFC and EO CBD content (Dp around 0.2 and 0.1 for TFC and CBD, respectively). The presence of two separate areas where D is higher than 0 was due to the uncommon behavior of TFC, which showed two maxima at long and short ET (Fig. 4).

The model validation was performed by operating two further extractions, V1 and V2, selecting the experimental parameters as those assuring peaks in the two different areas where D was higher than 0. Notably, the V1 run represents the optimized extraction since it possesses the highest D value among all the possible ones. The applied experimental conditions, the D values, the predicted values and the 95% interval of predictions are reported in Table 3. The results obtained in the validation runs were close to the predicted ones and always within the limit of 95% interval of prediction (Supporting Information Fig S4), indicating the reliability of the model obtained with the DoE analysis.

**Table 3:** Applied experimental conditions, D values, predicted values, and 95% interval of predictions of the validation runs V1 and V2

Validation run	MAE conditions			Composite desirability	Responses	Predicted value	95% interval of prediction
	Microwave power (W g^−1^)	Extraction time (min)	Added water (%)				
V1	1.5	160.5	55.4	0.68	EO yield	0.55	0.38–0.72
					EO CBD	4.7	3.1–6.3
					AE yield	870	547–1194
					AE TPC	109	93–127
					AE TFC	73	53–92
					AE DPPH	83	61–105
V2	1.5	79.2	60.6	0.42	EO yield	0.48	0.32–0.63
					EO CBD	3.2	1.6–4.7
					AE yield	978	647–1309
					AE TPC	118	101–136
					AE TFC	68	51–85
					AE DPPH	76	54–97

### LC‐DAD‐MS^*n*^ analysis of AE and HE

Run V1 represented the optimized extraction using the MAE‐based developed method having the highest D value. In order to support a possible application of AE and HE on an industrial level, a comprehensive HPLC‐DAD‐MS analysis was performed to highlight the content of bioactive compounds.

The HPLC‐DAD chromatogram of AE obtained in this run showed several peaks with UV spectra ascribable to phenolic derivatives supporting the presence of flavonoid and gallic acid derivatives (Supporting Information Fig S5). Compound identity was established using reference compounds and literature data; furthermore, annotation of other derivatives was obtained comparing our data with the literature, as indicated in Table 4. The total amount of flavonoids and other phenolic derivatives in the sample was 78.38 and 8.87 mg g^−1^, respectively. As previously reported, luteolin glucuronide (*m*/*z* 461) and apigenin glucuronide (*m*/*z* 445) were the most abundant species.^29^ Other apigenin and luteolin derivatives were also detected, along with gallocatechin, which were all present in notable amounts. HPLC‐MS^*n*^ in positive ion mode was used to evaluate the presence of cannabinoids. In the AE only CBD was quantifiable, while other cannabinoids were below the limit of detection. The amount of CBD was 0.85 mg g^−1^. The results revealed that AE obtained using the optimized MAE conditions could be a source of bioactive phenolic constituents, notably glycosidic flavones, with phytocannabinoids being almost absent. The abundance of these bioactive compounds may explain the noteworthy values obtained in terms of TPC (99 ± 8.9 mg GAE g^−1^_AE_), TFC (78 ± 4.7 mg rutin equiv. g^−1^_AE_), and DPPH radical scavenging activity (92 ± 5.5 mg Trolox equiv. g^−1^_AE_).

**Table 4:** Constituents of AE from run V1 as determined by LC‐DAD‐MS^*n*^ analysis

Constituent	Retention time (min)	[M + H]^−^	MS^2^	MS^3^	Concentration (mg g^−1^)	Reference
*Flavonoids*						
Apigenin 6,8‐di‐*C*‐glucoside^a^	8.6	593	503 473 383 353	383 353	5.99 ± 0.08	30
Luteolin di‐*C*‐hexoside	9.2	609	489 429 357 327	299 284	6.94 ± 0.06	31
Luteolin *C*‐(hexoside‐*O*‐rhamnoside)	9.6	593	473 429 357 327	299 284	4.61 ± 0.05	31
Vitexin‐2″‐*O*‐glucoside^a^	9.7	593	473 413 293	293	7.22 ± 0.07	32
Apigenin *C*‐(hexoside‐*O*‐rhamnoside)	10.1	577	457 413 293	293	1.72 ± 0.02	31
Eriodictyol‐7‐*O*‐glucoside^a^	10.6	449	269	251 225 209	3.77 ± 0.02	33
Luteolin‐7‐*O*‐glucuronide^a^	10.9	461	285 381 357 327	241 217 199 175 151	31.10 ± 0.10	31
Apigenin glucuronide	11.9	445	269	225 183 151	15.43 ± 0.08	31
*O*‐Methyl luteolin glucuronide	12.4	475	299	284	0.62 ± 0.01	31
Total					78.38	
*Other phenolics*						
(−)‐Gallocatechin	1.7	305	175	147 131	2.70 ± 0.03	34
Caffeoyl‐*O*‐hexoside	1.8	341	179 161 143		2.72 ± 0.04	34
Protocatecuic acid hexoside	6.1	315	153	109	0.79 ± 0.01	35
Unidentified	12.2	551	389 371 345 327	327 317 301	2.67 ± 0.02	
Total					8.87	
*Organic acids*						
Citric acid^a^	2.5	191	173 111	67	2.15 ± 0.03	
*Cannabinoids*						
Cannabidiol^a^	28.3	315	259 233 193 135 123	231 217 189 161	0.85 ± 0.01	

^a^ Indicates identification with reference standards.

In order to evaluate the presence of cannabinoids, the HE from residual biomass obtained in run V1 was subjected to the same analysis as the AE. The results are summarized in Table 5, and the HPLC‐DAD chromatogram is shown in Supporting Information Fig S6. None of the phenolic constituents that were detected in AE were present in HE, which was expected because the hexane extraction of phenolics and their glycosylated forms is highly unlikely due to solubility issues. On the other hand, HE presented a significant amount of cannabinoids, CBD being the most abundant one. The residual material, although subjected to MAE, still represents a significant source of cannabinoids worthy of further exploitation on an industrial level. Literature data indicate a medium value of 2–3% (w/w) of CBDA, and around 0.5% (w/w) of CBD for Futura 75.^39^ Thus our results indicated that mostly decarboxylated and partially non‐decarboxylated cannabinoids remained in the plant material after MAE. Notably, CBD was by far the predominant compound (160.5 mg g^−1^). Other minor cannabinoids occurring in the free form were cannabinol (15 mg g^−1^), a tetrahydrocannabinol isomer (9.7 mg g^−1^) and hydroxycannabidiol (3.9 mg g^−1^). The HPLC analyses showed that MAE did not completely decarboxylate all acid forms of cannabinoids since cannabidiolic acid (14.4 mg g^−1^), its derivative (4.3 mg g^−1^), cannabidivarinic acid (3.0 mg g^−1^) and hydroxycannabidivarinic acid derivative (2.4 mg g^−1^) were detected in the extract (Table 5). Notably, the ratio of cannabinoids/cannabinoid acids was 7.5 in the hexane extract obtained from the residual biomass, while the same ratio obtained from untreated plant material was mostly <0.1, with large amounts of acid forms.

**Table 5:** Constituents of HE from run V1 as determined by LC‐DAD‐MS^*n*^ analysis

Constituent	Retention time (min)	[M + H]^+^	MS^2^	MS^3^	Concentration (mg g^−1^)	Reference
*Cannabinoids (neutral forms)*						
Hydroxy‐ tetrahydrocannabinol	24.8	331	313 273 221 205 193 181 133	271 243 231 193	1.90 ± 0.05	36
Unidentified cannabinoid	25.6	315	259 233 193 135 123	231 217 189 161	3.04 ± 0.07	
Cannabidivarin^a^	26.1	287	231 205 193 165 153 135		3.37 ± 0.08	37
Hydroxy‐cannabidiol	27.8	331	313 193	271 257 243 231 193	3.85±0.06	36
Cannabidiol^a^	28.3	315	259 233 193 135 123	231 217 189 161	160.45 ± 0.11	
Cannabinol^a^	29.7	311	293 241 223	223 208 195	14.95 ± 0.09	37
Tetrahydrocannabinol isomer 1	30.3	315	259 233 193 135 123	231 217 189 161	2.74±0.04	37
Tetrahydrocannabinol isomer 2	30.7	315	259 233 193 135 123	231 217 189 161	9.70 ± 0.08	37
Cannabicyclol	31.3	315	259 233 193 135 123	231 217 189 161	0.09 ± 0.01	37
Total					200.10	
Unidentified	30.9	593	533	461 433 477 417	9.78 ± 0.08	
*Cannabinoids (acid forms)*						
Hydroxy‐cannabidivarinic acid derivative	22.3	347	329	311 205	2.38 ± 0.03	
Cannabidivarinic acid	23.5	329	311	268 173	3.04 ± 0.03	31
Hydroxy‐cannabidivarinic acid derivative	24.1	345	327 309	309 285	1.36 ± 0.02	
Hydroxy‐cannabidivarinic acid derivative	24.6	345	327 285 271 219 191		0.85 ± 0.01	
Cannabidiolic acid^a^	27.7	357	339	324 295 271 227	14.35 ± 0.09	
Cannabidiolic acid derivative	29.0	525	339	295 271 227	4.28 ± 0.06	
Total					26.25	
*Geranyl flavones*						
Cannflavin C^a^	25.0	435	—	—	0.01 ± 0.01	38
Cannflavin A^a^	26.9	435	420 351	336 323 309	0.26 ± 0.01	38
Total					0.27	

^a^ Indicates identification with reference standards.

In conclusion, the byproduct generated during MAE can be reused to extract and purify cannabinoids, which are mostly present in decarboxylated forms, with CBD as the most representative compound. Besides, an important fraction of polyphenols such as glycosidic flavones can be recovered. This is definitely an added value for the pharmaceutical and cosmetic markets.

### Biological assays (α‐glucosidase, AGEs, lipase and superoxide radicals inhibition) of AE and HE

HPLC‐DAD‐MS analysis showed a significant number of phenolic constituents and cannabinoids in AE and HE, respectively. Literature data suggest that these compounds possess enzyme inhibition capacity.^40^, ^41^ For this reason, it was decided to subject AE and HE to different bioassays to evaluate their biological properties in terms of antidiabetic and anti‐obesity potential. In this sense, these extracts were tested as enzyme (α‐glucosidase and lipase) inhibitors but also for their capacity to decrease the formation of advanced glycation end products (AGEs) and superoxide radicals.

**Figure 7:**
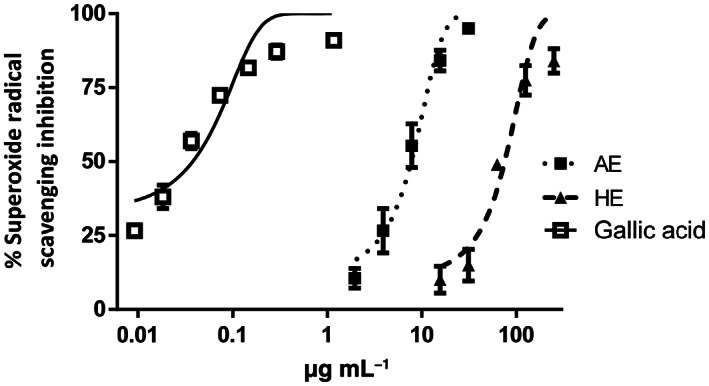
Radical scavenging activity against superoxide radicals generated by the xanthine/xanthine oxidase system.

Table 6 provides the results of the IC_50_ values for the different assays; it can be observed that AE was the best extract in terms of α‐glucosidase inhibition and as a superoxide radical scavenger. Both extracts (AE and HE) had the ability to inhibit AGE formation, but none of them could act as lipase inhibitors (Fig. 7). It is noteworthy that AE is more potent than the reference drug acarbose as a glucosidase inhibitor; this is probably due to the specific phenolic profile of this extract, particularly in terms of flavonoids. Indeed, the studies by Proença *et al*. showed the efficacy of flavonoids in inhibiting α‐glucosidase.^40^

**Table 6:** Inhibitory activities of the extracts (IC_50_ values presented in μg mL^−1^) in the α‐glucosidase, superoxide radical scavenging and AGE assays^a^

	α‐Glucosidase	Superoxide radical scavenging	AGE
AE	164.1±10.08	7.06±2.13	169.1
HE	—	63.84±2.86	183.5±71.61
Acarbose	294.34±34.18	—	—
Gallic acid	—	0.034±0.016	—
AMG	—	—	63.42±14.46

^a^ Data were represented as mean ± standard deviation (*n* = 3); —, not determined due to very low activity. Lipase inhibition was not detected.

Natural α‐glucosidase inhibitors have already been detected in *C. sativa* samples.^41^, ^42^, ^43^, ^44^ In particular, Ma *et al*.^41^ reported the moderate inhibitory effect of CBD on α‐glucosidase, while the same activity was ascribed to oligopeptides from hemp seed protein by Ren *et al*.^44^ However, this is the first time that hemp by‐products of EO extraction are presented as interesting substances containing bioactive compounds with antidiabetic and antioxidant potential. In the AGE experiment, both extracts had the ability to inhibit the formation of advanced glycation end products, an activity never reported before. It can here be deduced that polyphenolic compounds detected in AE are responsible for these activities; conversely, HE could not inhibit α‐glucosidase, probably since phytocannabinoids do not act as strong enzyme inhibitors, at least in the α‐glucosidase and pancreatic lipase assays. Orlistat is a well‐known drug acting as pancreatic lipase inhibitor in overweight and obese patients; although orlistat is not a polyphenolic compound, certain flavonoids such as quercetin have demonstrated the capacity to inhibit pancreatic lipase.^45^ However, in this case AE does not contain quercetin, which could explain in part the lack of activity and the fact that most of the flavonoids in AE are in form of glycosides, avoiding enzyme–compound interactions in pancreatic lipase.^45^ Hemp hydrophilic compounds with radical scavenging, AGEs and glucosidase inhibitory properties might be very useful for the treatment of metabolic disorders.

## CONCLUSIONS

The MAE process of hemp EO and its by‐products (the aqueous residue AE and the residual biomass HE extracts, respectively) were studied and optimized using a DoE statistical approach. The experimental parameters applied, MP, ET and W% influenced in a different manner the quality and quantity of the obtained products, with the exception of HE. All the studied features, yields, CBD content in the EO, and the polyphenols, flavonoids and radical scavenging activity of AE were optimized (maximized) operating at high MP and ET using an amount of water in the range of 50–60%. The optimized conditions were experimentally validated, and the two obtained by‐products were further analyzed in terms of composition and biological activity. The AE was rich in phenolic compounds, especially glycosidic flavones, and acted as superoxide radical scavenger, AGEs and an α‐glucosidase inhibitors. The latter activity is particularly promising since the AE performed markedly better than the reference compound acarbose. The HE showed a relevant content of cannabinoids, especially in the decarboxylated forms, with CBD as the predominant one. It was able to reduce AGE formation as well as the scavenging superoxide radicals activity, even in the less extent than AE.

The results of this work support the valorization of industrial hemp EO and its by‐products, obtained by a sustainable and eco‐friendly extraction method, through an almost waste‐free approach. Cannabinoids as well as other valuable bioactive compounds such as glycosidic flavones may be recovered from the residues of the EO extraction, representing interesting substances in the pharmaceutical, cosmetic and nutraceutical fields. In addition to the known applications, this study also suggests the possible use of AE as adjuvant in the treatment of metabolic disorders such as type 2 diabetes and obesity.

## Supporting information


**Appendix S1:** Supporting Information
