# Supplementary material for: Green extraction of hemp (Cannabis sativa L.) using microwave method for recovery of three valuable fractions (essential oil, phenolic compounds and cannabinoids): a central composite design optimization study
Source: J Sci Food Agric. 2022 May 18;102(14):6220–35. doi: 10.1002/jsfa.11971 (PMC9790304; doi:10.1002/jsfa.11971)
Supplement: Supplementary file 1 — Appendix S1: Supporting Information [file JSFA-102-6220-s001.docx]

**SUPPLEMENTARY MATERIAL OF**

**Green extraction of hemp *(Cannabis sativa* L.) using microwave method for recovery of three valuable fractions (essential oil, phenolic compounds and cannabinoids): a central composite design optimization study**

Eugenia Mazzara^a^, Riccardo Carletti^a^, Riccardo Petrelli^a^, Ahmed M. Mustafa^a,b^, Giovanni Caprioli^a^, Dennis Fiorini^c^, Serena Scortichini^c^, Stefano Dall’Acqua^d^, Sonia Nuñez^e^, Victor López^e^, Valtcho D. Zheljazkov^f^, Giulia Bonacucina^a^, Filippo Maggi^a*^, Marco Cespi^a^

^a^School of Pharmacy, University of Camerino, Camerino, Italy

^b^Department of Pharmacognosy, Faculty of Pharmacy, Zagazig University, Zagazig, Egypt

^c^School of Science and Technology, University of Camerino, Camerino, Italy

^d^Department of Pharmaceutical and Pharmacological Sciences, Natural Product Laboratory, University of Padova, Padova, Italy

^e^Department of Pharmacy, Faculty of Health Sciences, Universidad San Jorge, Villanueva de Gállego, Zaragoza, Spain

^f^Crop and Soil Science Department, 3050 SW Campus Way, Oregon State University, Corvallis, OR 97331, U.S.A.

Table ST1. Chemical composition of hemp EOs obtained by MAE runs No 1, 8, 10.

| **N°** | **Component^a^** | **RI^b^** | **RI lit.^c^** | **Relative peak area (%)** | | | **ID^d^** | | |
| --- | --- | --- | --- | --- | --- | --- | --- | --- | --- |
|  |  |  |  | **MAE** | | |  |  | |
|  |  |  |  | **run 1** | **run 8** | **run 10** |  |  | |
| 1 | 5,5-dimethyl-1-vinylbicyclo[2.1.1]hexane | 919 | 920 | 0.3 | 0.2 | 0.2 | RI,MS | |  |
| 2 | α-thujene | 927 | 924 | 0.1 | tr^e^ | 0.1 | RI,MS | |  |
| 3 | α-pinene | 933 | 932 | 21.8 | 19.5 | 20.6 | Std | |  |
| 4 | camphene | 948 | 946 | 0.2 | 0.2 | 0.2 | Std | |  |
| 5 | β-pinene | 976 | 974 | 5.6 | 5.2 | 5.6 | Std | |  |
| 6 | myrcene | 992 | 988 | 17.6 | 16.7 | 16.8 | Std | |  |
| 7 | α-phellandrene | 1004 | 1002 | 0.2 | 0.2 | 0.3 | Std | |  |
| 8 | δ-3-carene | 1010 | 1008 | 0.6 | 0.6 | 1.0 | Std | |  |
| 9 | α-terpinene | 1017 | 1014 | 0.1 | 0.1 | 0.2 | Std | |  |
| 10 | *p*-cymene | 1025 | 1020 | 0.2 | 0.2 | 0.2 | Std | |  |
| 11 | limonene | 1029 | 1024 | 2.3 | 1.8 | 2.1 | Std | |  |
| 12 | 1,8-cineole | 1031 | 1026 | 0.2 | 0.1 | 0.1 | Std | |  |
| 13 | (*Z*)-β-ocimene | 1039 | 1032 | 0.3 | 0.3 | 0.3 | Std | |  |
| 14 | (*E*)-β-ocimene | 1049 | 1044 | 4.0 | 4.3 | 4.2 | Std | |  |
| 15 | γ-terpinene | 1059 | 1054 | 0.1 | 0.1 | 0.2 | Std | |  |
| 16 | terpinolene | 1088 | 1086 | 8.2 | 8.9 | 12.4 | Std | |  |
| 17 | (*E*)-caryophyllene | 1421 | 1417 | 21.9 | 22.0 | 21.0 | Std | |  |
| 18 | α-*trans*-bergamotene | 1437 | 1432 | 1.8 | 1.7 | 1.4 | RI,MS | |  |
| 19 | α-humulene | 1455 | 1452 | 6.6 | 6.9 | 6.4 | Std | |  |
| 20 | (*E*)-β-farnesene | 1458 | 1454 | 1.5 | 1.4 | 1.1 | Std | |  |
| 21 | *allo*-aromadendrene | 1463 | 1458 | 0.7 | 0.8 | 0.5 | RI,MS | |  |
| 22 | selina-4,11-diene | 1486 | 1476 | 0.2 | 0.2 | 0.1 | RI,MS | |  |
| 23 | β-selinene | 1488 | 1489 | 0.9 | 1.0 | 0.6 | RI,MS | |  |
| 24 | α-selinene | 1497 | 1498 | 0.8 | 0.9 | 0.5 | RI,MS | |  |
| 25 | δ-cadinene | 1526 | 1522 | 0.1 | 0.1 |  | RI,MS | |  |
| 26 | selina-4(15),7(11)-diene | 1537 | 1544 | 0.5 | 0.3 | 0.2 | RI,MS | |  |
| 27 | selina-3,7(11)-diene | 1544 | 1538 | 1.1 | 0.8 | 0.6 | RI,MS | |  |
| 28 | caryophyllene oxide | 1586 | 1583 | 1.3 | 3.2 | 1.5 | Std | |  |
| 29 | humulene epoxide II | 1612 | 1608 | 0.2 | 0.7 | 0.2 | RI,MS | |  |
| 30 | cannabidiol | 2430 | 2430 | tr | 0.5 | 0.2 | Std | |  |
| 31 | cannabichromene | 2438 | 2440 |  | tr | tr | RI,MS | |  |
|  |  |  |  |  |  |  |  | |  |
|  | Total identified (%) |  |  | 99.2 | 99.1 | 98.8 |  | |  |

^a^ Order of elution obtained by an HP-5MS column (30m x 0.25 mm, 0.1 µm).

^b^ Linear retention index according to Van den Dool and Kratz (1963).

^c^ RI are from ADAMS and/or NIST 17 and FFNSC3 libraries.

^d^ Identification method: Std, comparison with analytical standard; RI, coherence of the calculated RI with those reported in ADAMS, NIST 17 and FFNSC3 libraries. MS, mass spectrum overlapping with those recorded in ADAMS, NIST 17, WILEY 275 and FFNSC3 libraries.

^e^ Traces, relative %<0.1.

Table ST2. Coefficient and residual analysis of the models used for the optimization step

| Responses | Analysis of coefficient | Residual analysis |
| --- | --- | --- |
| EO Yield (ml/Kg) | 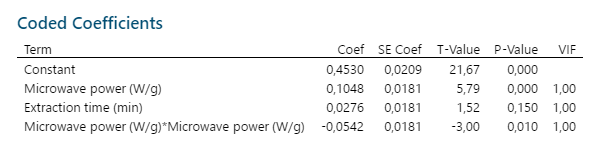 |  |
| EO CBD (g/100 g) | 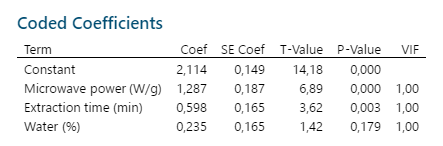 |  |
| AE Yield (mg/100 g) | 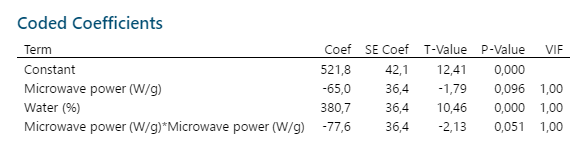 |  |
| AE TPC (mg GAE/g ex) | 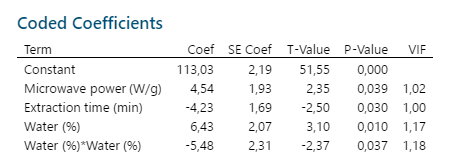 |  |
| AE TFC (mg rutin eq./g ex) | 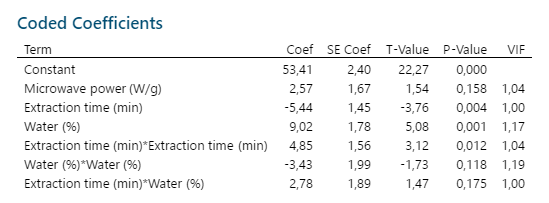 |  |
| DPPH (mg TROLOX eq./g ex) | 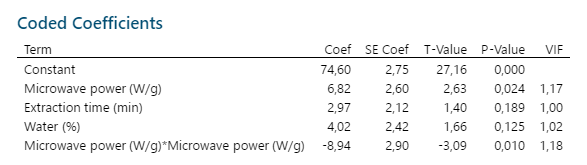 |  |


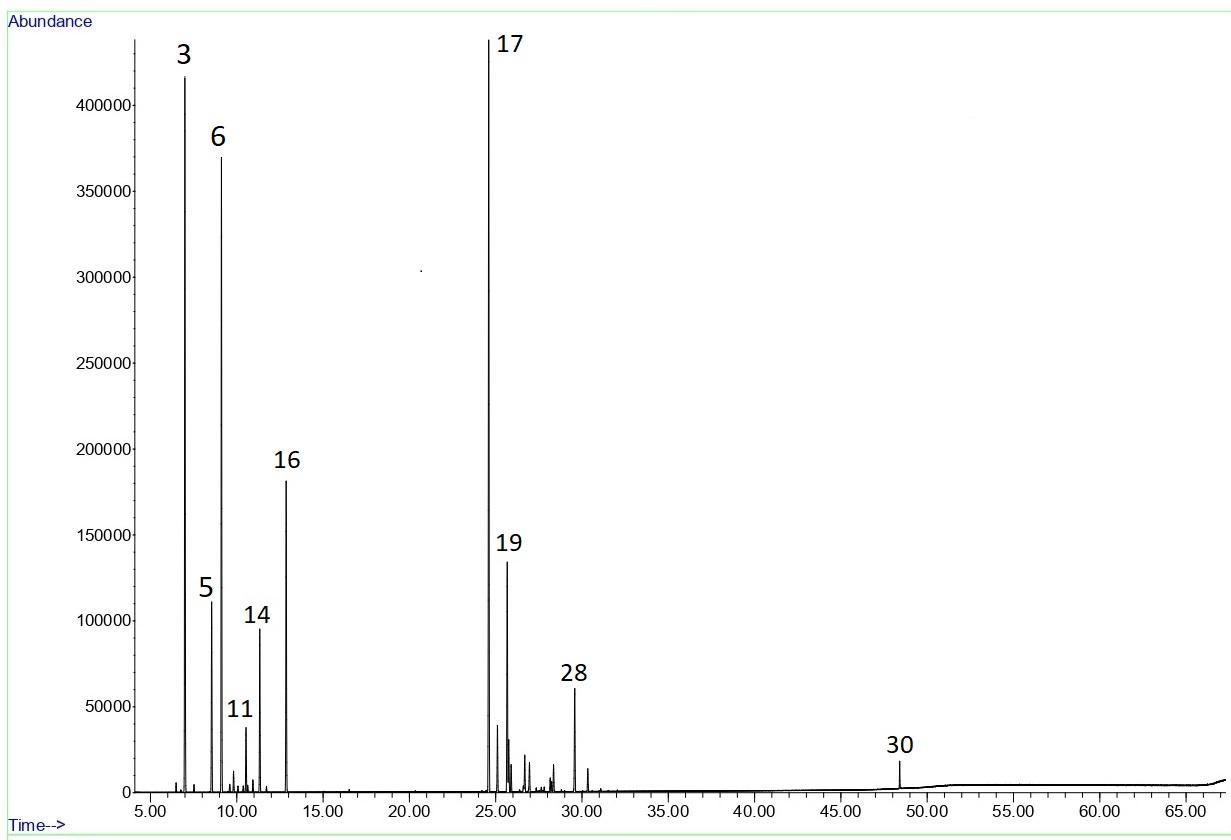


Figure SF1. GC-MS chromatogram of the EO (run No 8); peak numbering refers to Table ST1.


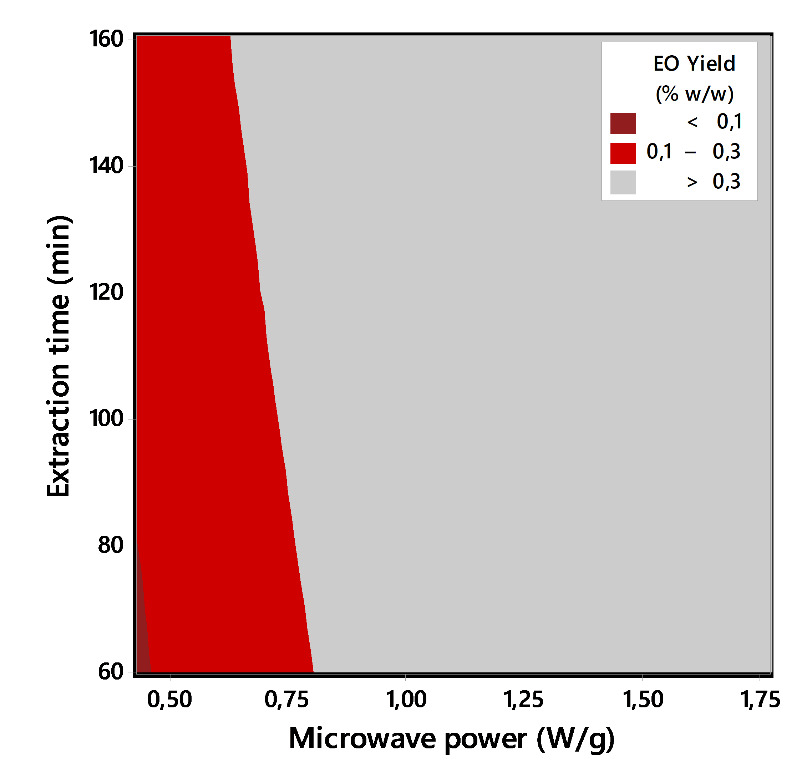


Figure SF2. Contour plot of the EO yield as a function of microwave power and extraction time as predicted by the model for the yield. The red area highlights the experimental conditions necessary to obtain yield values corresponding to those reported in the literature*.*

Figure SF3. GC-FID chromatogram of the HE from run No 8; the main peak refers to CBD.
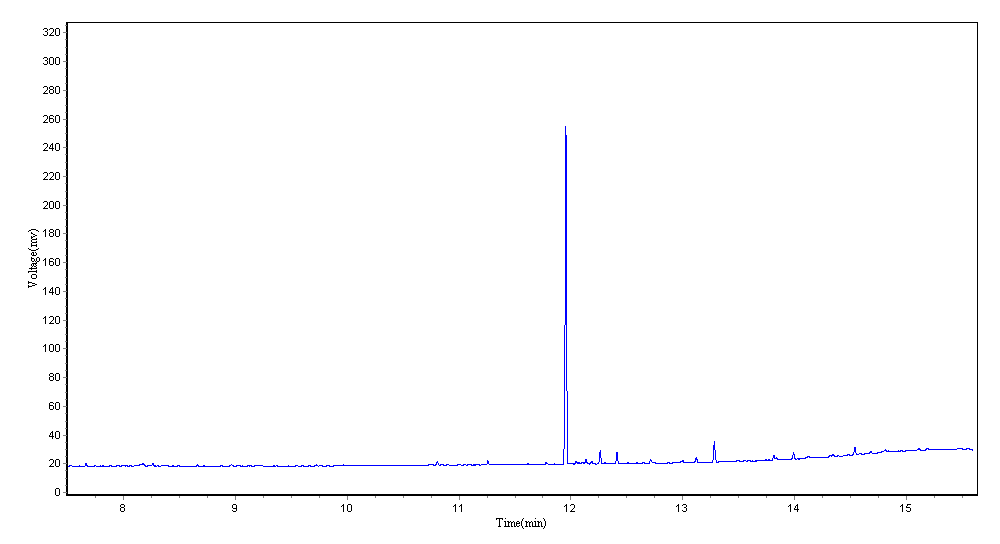


Figure X: GC-FID chromatogram of residual biomass hexane extract from MAE run No 8.


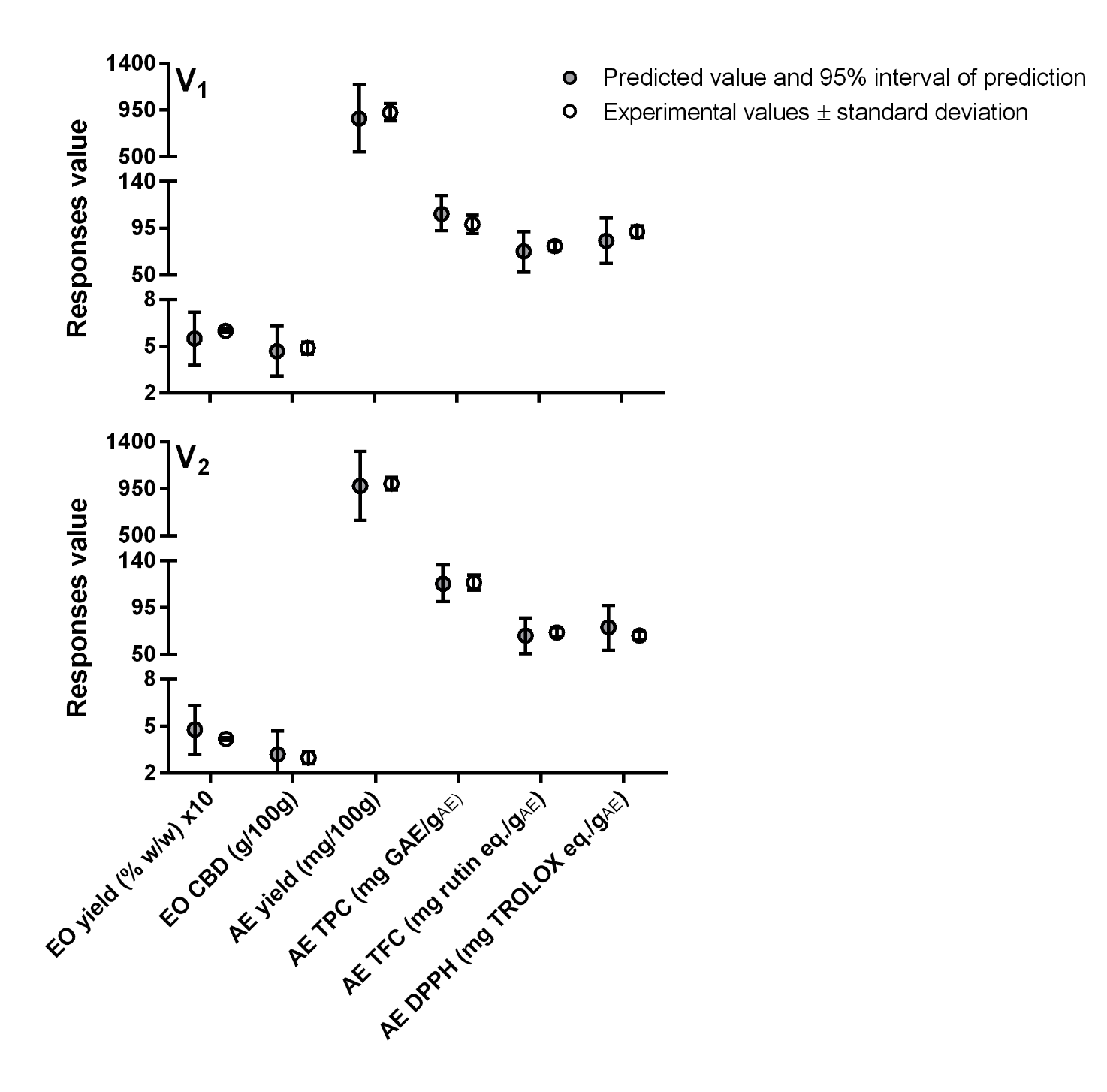


Fig. SF4. Comparison of the results obtained from the runs V1 and V2 against the desirability predicted values.


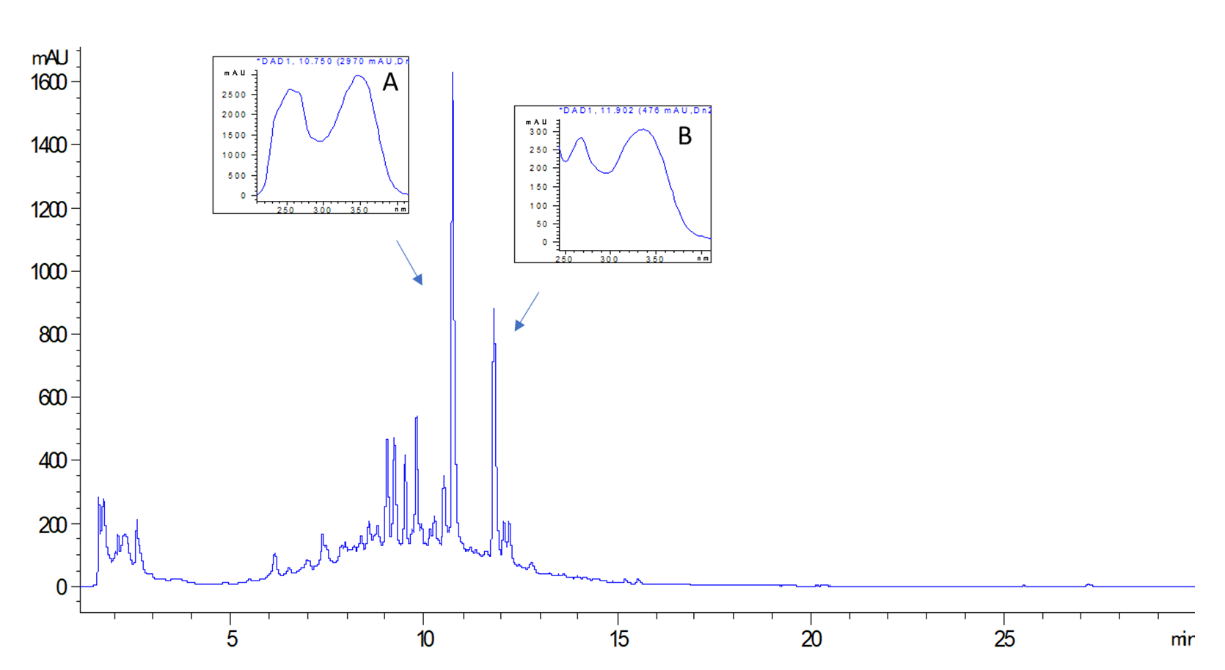


Fig. SF5. HPLC-DAD chromatogram of AE from run V1 at 280 nm. A and B represent UV spectrum of the main constituents, namely luteolin glucuronide and apigenin glucuronide, respectively.


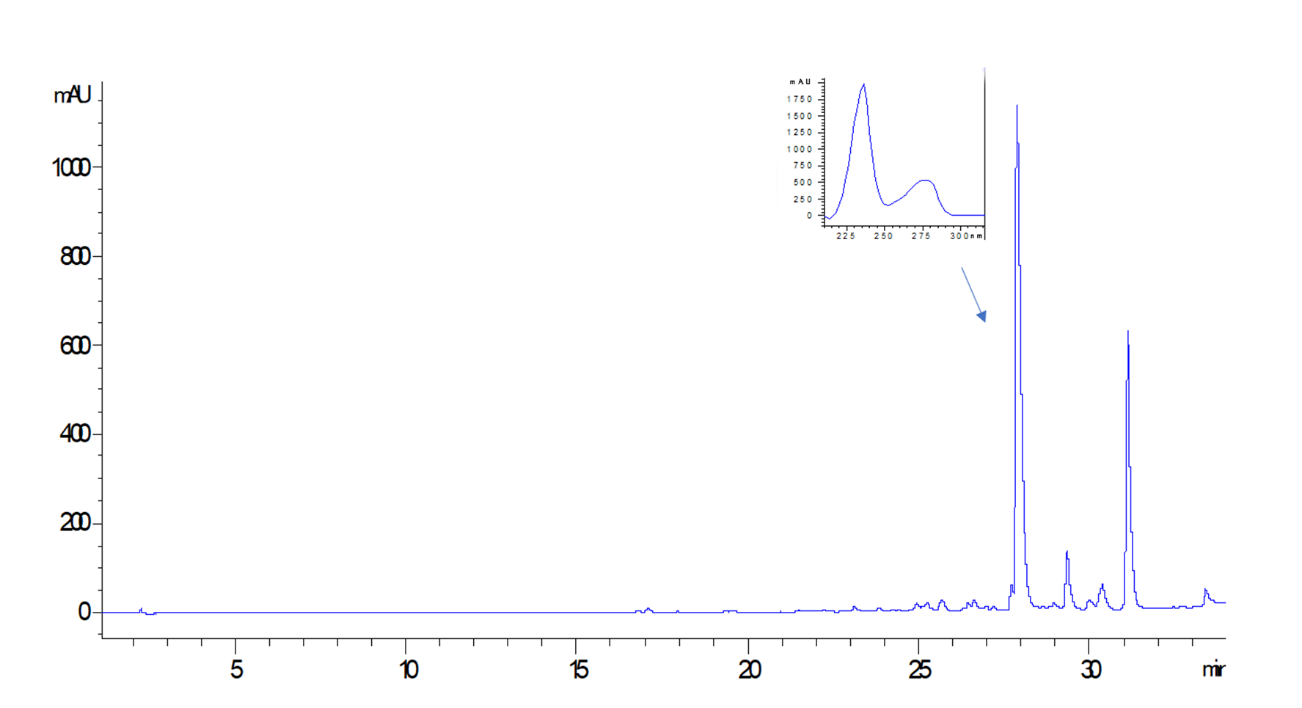


Fig. SF6. HPLC-DAD chromatogram of HE from run V1 at 280 nm. UV spectrum of CBD was reported.
